# Immunogenicity of Varicella Zoster Virus DNA Vaccines Encoding Glycoprotein E and Immediate Early Protein 63 in Mice

**DOI:** 10.3390/v14061214

**Published:** 2022-06-02

**Authors:** Jie Liu, Junyang Lin, Linjun Cai, Jie Sun, Xue Ding, Cenrong Wang, Yanchun Wu, Xiaoling Gao, Weiheng Su, Chunlai Jiang

**Affiliations:** 1 National Engineering Laboratory for AIDS Vaccine, School of Life Sciences, Jilin University, Changchun 130012, China; jieliu18@mails.jlu.edu.cn (J.L.); lin3226544@163.com (J.L.); linjun_cai@jlu.edu.cn (L.C.); sunjie20@mails.jlu.edu.cn (J.S.); dingxue20@mails.jlu.edu.cn (X.D.); wangcr20@mails.jlu.edu.cn (C.W.); 2 Key Laboratory for Molecular Enzymology and Engineering of the Ministry of Education, School of Life Sciences, Jilin University, Changchun 130012, China; 3 Animal Experiment Center, Changchun BCHT Biotechnology Co., Changchun 130012, China; wuyanchun@bchtpharm.com (Y.W.); gaoxiaoling2022@163.com (X.G.)

**Keywords:** herpes zoster, glycoprotein E, immediate early protein 63, DNA vaccines, cell-mediated immunity

## Abstract

Herpes zoster (HZ) is caused by the reactivation of latent varicella-zoster virus (VZV) from the sensory ganglia due to aging or immunosuppression. Glycoprotein E (gE) is a widely used vaccine antigen for specific humoral and cellular immune responses. Immediate early protein 63 (IE63) is expressed during latency, suggesting that it is a potential antigen against HZ reactivation. In this study, HZ DNA vaccines encoding gE, IE63, IE63-2A-gE (where 2A is a self-cleaving sequence), or IE63-linker-gE were developed and investigated for immunogenicity in mice. The results showed that each HZ DNA vaccine induced VZV-specific antibody production. The neutralizing antibody titer elicited by IE63-2A-gE was comparable to that elicited by gE or live attenuated HZ vaccine (LAV). IE63-2A-gE-induced gE or IE63-specific INF-γ^+^ T cell frequencies in splenocytes were comparable to those of LAV. Furthermore, IE63-2A-gE, gE, or IE63 led to a significant increase in IFN-γ (IE63 stimulation) and IL-2 (gE stimulation) secretion compared to LAV, showing a Th1-biased immune response. Moreover, IE63-2A-gE and gE induced cytotoxic activity of CD8^+^ T cells compared to that of LAV. This study elucidates that the IE63-2A-gE DNA vaccine can induce both humoral and cell-mediated immune responses, which provides a candidate for the development of an HZ vaccine.

## 1. Introduction

Varicella-zoster virus (VZV) is a highly contagious virus belonging to the Alphaherpesvirinae subfamily [1]. Primary VZV infection causes varicella, which usually occurs in childhood and causes fever and herpes. VZV can remain dormant in the dorsal root ganglia after infection, while reactivation of the latent virus from sensory ganglia results in Herpes zoster (HZ) [2]. The incidence of HZ is approximately 0.4% in those aged 50–60 years and significantly increases to over 1% in those over the age of 80 years [3,4,5]. HZ is usually followed by chronic and debilitating pain called post-herpetic neuralgia (PHN), which can last several months after the rash, especially in the elderly [1,6,7]. The humoral immune response elicited by the VZV vaccine can effectively prevent the incidence of varicella. In contrast, lower VZV-specific cell-mediated immunity (CMI) is correlated with HZ onset and a high incidence of PHN [8,9,10,11]. Therefore, the ability to elicit VZV-specific CMI is thought to be important for the prevention of VZV infection and reactivation.

Currently, two licensed vaccines are available to prevent HZ: a live attenuated VZV vaccine, Zostavax^®^, and a recombinant subunit vaccine, Shingrix^®^. Zostavax^®^ has been licensed since 2006 and contains over ten times the amount of VZV antigen compared to the attenuated varicella vaccine. Although Zostavax^®^ can prevent HZ and PHN in adults, its efficacy decreases with age, especially in those aged ≥70 years [12]. Shingrix^®^ contains VZV gE and the AS01B adjuvant system, which elicits a remarkably high vaccine efficacy in the elderly after two injections [13,14]. Owing to its high protection against shingles regardless of age, the FDA has recently recommended Shingrix^®^ as an alternative vaccine. Studies have shown that Shingrix^®^ can elicit high levels of antibodies and strong gE-specific CD4^+^ T cell responses, especially high IFN-γ [15], implying the significance of cellular immunity in preventing HZ.

VZV gE is the most abundant and immunogenic VZV glycoprotein. VZV gE is the major neutralizing antibody-inducing antigen that contains B-cell and CD4^+^ T-cell epitopes [16]. Therefore, VZV gE is the most commonly chosen antigen for HZ vaccines to induce humoral and cellular immune responses. Previous studies showed an enhanced antibody response when mice were immunized with a truncated form of gE [17,18]. Therefore, we chose the extracellular domain of gE as the antigen in this study. IE63 (immediate early protein 63) is a tegument protein in VZV. Studies indicated that IE63 is expressed in infected human and rat sensory ganglia during the latency period. Grinfeld et al. reported IE63 expression in all ganglia from 10 donors [19]. Lungu et al. detected IE63 protein expressed in all ganglia from three donors [20]. Kennedy et al. detected nine of 35 donors positive for IE63 [21]. Gershon et al. detected IE63 protein in enteric neurons with latent VZV infection in a guinea pig model [22]. Mahalingam et al. reported two of 9 subjects positive for IE63 in ganglia [23]. A study by Zerboni et al. only found one of 18 individuals positive for IE63 and in less than 2.8% of neurons [24]. Although the expression of IE63 varied among different studies, IE63 is still considered as a potential latency protein. In one study, IE63-specific T-cell responses were significantly impaired in patients with subclinical VZV reactivation, suggesting that T-cell responses to IE63 are important for controlling VZV replication [25]. Louise Jones et al. implicated IE63-specific CD4^+^ T cells is important in the control of viral reactivation [26]. Janet et al. demonstrate that poly-functional IE63-CD4^+^ T cells may contribute toward Zostavax^®^ efficacy [27]. Therefore, IE63 would be a reasonable candidate for a T-cell antigenic target for both control of viral replication and induction of immune responses and appears to be a suitable vaccine candidate for developing HZ vaccines [26]. Michele et al. evaluated the immunogenicity of a recombinant gE–IE63 subunit vaccine, mainly focusing on humoral immune responses [28]. However, more comprehensive evaluations of cell-mediated immune responses induced by vaccines containing IE63 antigen are needed.

Since its discovery in the 1990s, DNA vaccination has been considered capable of inducing both humoral and cell-mediated immune responses while showing advantages in production, stability, and storage [29]. Therefore, DNA vaccines have been suggested as both preventative and therapeutic approaches, especially for infectious diseases and cancers [30].

In this study, we tested two combinations of VZV gE and IE63 as DNA vaccine antigens. One encodes a fusion protein, comprising an IE63 and a downstream extracellular domain of gE. The other encodes an IE63 and fuses a downstream extracellular domain of gE, connected by mouth and foot disease virus P2A self-cleaving sequence that self-cleaves during the translation progress [31]; this design can express separate IE63 and truncated gE proteins in cells. We assessed humoral and cell-mediated immunity in VZV-primed C57/6J mice and found that HZ DNA vaccines induced a VZV-specific antibody titer, Th1/Th2 cytokine secretion, and a high IFN-γ^+^ immune cell frequency.

## 2. Materials and Methods

### 2.1. Cells

293T and EL-4 cell lines were purchased from the American Type Culture Collection (Manassas, VA, USA; CCL-171). Cells were cultured in Minimum Essential Medium (MEM; Invitrogen, Carlsbad, CA, USA), supplemented with 10% fetal bovine serum, 100 µg/mL streptomycin, and 100 U/mL of penicillin, and maintained in a 5% CO_2_ atmosphere at 37 ℃.

### 2.2. DNA Plasmids

The genes encoding the truncated gE (1-545 aa) [17,18], full-length IE63, IE63-2A-gE (2A: GSGATNFSLLKQAGDVEENPGP), and IE63-linker-gE (linker: LQGGGSRGGG) were synthesized commercially by GenScript Biotech Corp. (Nanjing, China) and were inserted into the pcDNA3.1(+) vector for the expression of heterologous proteins in mammalian cells.

### 2.3. Protein Expression

For protein expression analysis, 293T cells were transfected with pcDNA3.1-gE, pcDNA3.1-IE63, pcDNA3.1-IE63-2A-gE, or pcDNA3.1-IE63-linker-gE. Forty-eight hours after transfection, cells and supernatants were obtained and analyzed using western blotting. After electrophoresis, proteins were transferred onto nitrocellulose membranes. Membranes were first incubated with a mouse-specific primary antibody against gE (ab272686; Abcam, UK) or rabbit polyclonal antibodies against IE63 (a gift from Dr. Paul R. Kinchington of University of Pittsburgh) for two hours and then with the secondary goat anti-mouse (115-035-003; Jackson ImmunoResearch Inc., West Grove, PA, USA) or goat anti-rabbit IgG-horseradish peroxidase (HRP) (111-035-045; Jackson ImmunoResearch Inc.) for 45 min. The nitrocellulose membrane was incubated with ECL luminescence reagent (Meilunbio, China, Cat. MA0186-1). Images were captured using an ECL system (Tanon, China).

### 2.4. Animals

Six- to eight-week-old SPF female C57BL/6J mice were purchased from Liaoning Changsheng Biotechnology Co. Ltd. (Liaoning, China). All mice used in this study were treated according to the Guide for the Care and Use of Laboratory Animals (Jilin University). All experimental procedures were reviewed and approved by the Animal Welfare and Research Ethics Committee of the Jilin University (Approval no. 2020-nsfc008).

### 2.5. Immunizations

Mice were divided into seven groups (five mice per group). Except for the mice in the gE (no prime) group, each mouse was primed with live attenuated VZV vaccine at a dose of 6300 PFU. Twenty-eight days after priming, mice were intramuscularly immunized three times at 2-week intervals with 50 μg pcDNA3.1-gE, pcDNA3.1-IE63, pcDNA3.1-IE63-2A-gE or pcDNA3.1-IE63-linker-gE, respectively, in 100 μL of phosphate-buffered saline (PBS), followed by in vivo electroporation using an electric pulser (TERESA, China) with each time DNA immunization. The negative control mice were immunized three times at 2-week intervals with 100 μL of PBS, and mice in the group vaccinated three times at 2-week intervals with a one-tenth dose of live attenuated VZV vaccine were used as the positive controls. Two weeks after the last immunization, the mice were euthanized by CO_2_ overdose, and their spleens were obtained.

### 2.6. Antibody Analysis

VZV gE and IE63 specific total immunoglobulin G (IgG) were detected in the mouse serum using ELISA. The 96-well ELISA plates were coated with VZV-gE (0.5 μg/well) or VZV-IE63 (0.5 μg/well) overnight at 4 °C. The plates were washed thrice with PBS containing 0.05% Tween 20 and blocked with 3% bovine serum albumin at 25 °C for one hour. The immunized mouse serum was serially diluted two-fold and added to each well, and HRP-conjugated goat anti-mouse IgG was added. 3,3′,5,5′-tetramethylbenzidine was then used for the color reaction, and 2 M H_2_SO_4_ was used for the termination reaction. Using a microplate reader, the optical density was measured at 450 nm. The optical density value of the highest dilution was 2.1-fold higher than that of the negative control at the same dilution and was used as the serum endpoint dilution titer.

A fluorescent antibody to membrane antigen (FAMA) was used to measure the production of neutralizing antibodies against VZV. V-Oka-infected MRC-5 cells were first incubated with two-fold serially diluted serum from 1:4 to 1:2048. Then, the samples were incubated with anti-mouse antibodies labeled with fluorescein isothiocyanate and pre-mixed with Evans blue. The results were observed under a fluorescence microscope (Olympus IX70, Tokyo, Japan).

### 2.7. Th1/Th2 Cytokine Detection

Mouse spleens were extracted under aseptic conditions for 14 days after the final immunization. The spleen cell suspensions were diluted to 10^7^ cells/mL and incubated with Roswell Park Memorial Institute (RPMI) 1649 medium (negative control), gE or IE63 protein (10 µg/mL), gE or IE63 peptides (10 µg/mL, 15-mer overlapping by 11-mer), and ConA (10 µg/mL, positive control) for 48 h. The supernatant was collected and analyzed for IFN-γ, IL-2, and IL-10 secretion using a Th1/Th2 Mouse Uncoated ELISA Kit (Cat. no. 88-7711-44) according to the manufacturer’s instructions. A standard curve was used to calculate the cytokine concentrations.

### 2.8. Interferon-γ ELISPOT Assays

Splenocyte suspensions (10^7^ cells/mL) were incubated with PRIM 1640 medium (negative control), gE protein (extracellular domain, 1–545 aa, 5 µg/mL), IE63 protein (full-length, 5 µg/mL), gE peptides (10 µg/mL, 15-mer overlapping by 11-mer), IE63 peptides (10 µg/mL, 15-mer overlapping by 11-mer), or ConA (5 µg/mL, positive control) for 24 h. The assay was performed using a mouse IFN-γ ELISpotPLUS kit (Cat. no. 3321-4HPW-10) according to the manufacturer’s instructions. The number of spots was determined using an ImmunoSpot S6 analyzer (Cellular Technology Limited, Shaker Heights, OH, USA).

### 2.9. In Vitro Cytotoxic T Cell Assay

Antigen-specific CD8^+^ T-lymphocyte immune responses were characterized using a cytotoxicity assay. Cytotoxicity assays were performed according to previously published procedures [32]. Briefly, EL-4 murine tumor cell lines as target cells were first labeled with gE or IE63 peptides at 37 °C and 5% CO_2_ for two hours. After washing twice with RPMI 1640 medium, gE^+^ EL-4, IE63^+^ EL-4, and EL-4 cells were labeled with different concentrations of carboxyfluorescein succinimidyl ester (CFSE) fluorescent dye. Splenocytes (5 × 10^5^) isolated from immunized mice were incubated at an effector-to-target ratio of 50:1 with target cells for 10 h at 37 °C and 5% CO_2_. Cytotoxicity was analyzed by flow cytometry, and antigen-specific lethality was calculated according to the following formula: cytotoxicity = [1 − (antigen^+^ EL-4 cells/unloaded cells from the immunized group)/(EL-4 cells/unloaded cells from the naïve group)] × 100%.

### 2.10. Statistical Analysis

All experiments were independently repeated twice. Values are expressed as mean ± standard deviation (SD). Statistical analysis was performed by one-way analysis of variance using GraphPad Prism version 9.0.0 (GraphPad Software, San Diego, CA, USA). Differences were considered significant at *p* < 0.05, with a 95% confidence level.

## 3. Results

### 3.1. Design and Expression of the HZ DNA Vaccines

**Figure 1:**
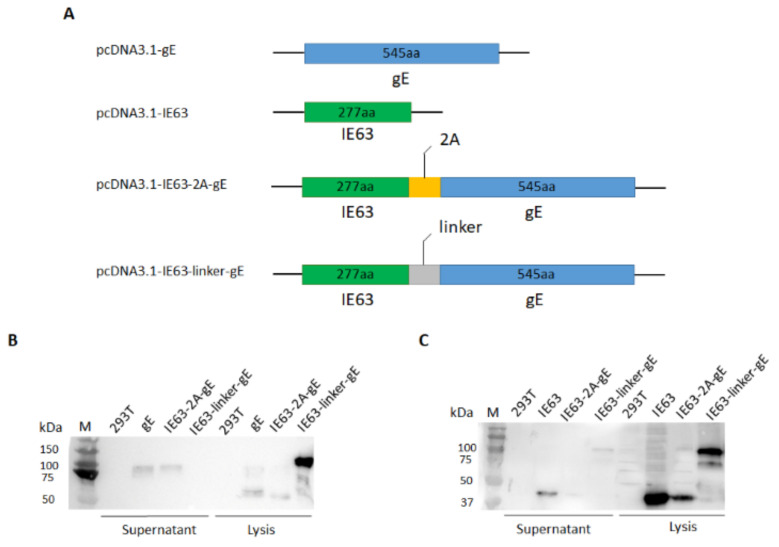
Design of plasmids and the expression in 293T cells detected by western blotting. (**A**). Schematics of the pcDNA3.1-gE, pcDNA3.1-IE63, pcDNA3.1-IE63-2A-gE and pcDNA3.1-IE63-linker-gE designs. (**B**). Expression of the gE protein in the supernatant and cell lysis was probed using mouse anti-gE monoclonal antibody. (**C**). Expression of the IE63 protein in the supernatant and cell lysis was probed using rabbit anti-IE63 polyclonal antibodies.

In this study, we analyzed the immunogenicity of VZV gE and IE63, alone or in combination, as DNA vaccine antigens and designed two different fusion proteins (Figure 1A). One of them encoded an IE63 and gE fusion protein connected by a linker sequence. The other was connected by a mouth and foot disease virus 2A self-cleaving sequence that self-cleaves during the translation process, which can express separate IE63 and truncated gE proteins in cells. To confirm the expression and secretion of antigen proteins in mammalian cells, recombinant plasmids were transfected into 293T cells, and cell lysates and supernatants were assayed using western blotting. When detected with the anti-gE antibody (Figure 1B) and anti-IE63 antibody (Figure 1C), the gE, IE63, IE63-2A-gE and IE63-linker-gE groups showed bands consistent with the predicted molecular weight. Thus, HZ DNA vaccines were successfully constructed and expressed.

### 3.2. HZ DNA Vaccines Induce VZV -Specific Humoral Immune Responses

**Figure 2:**
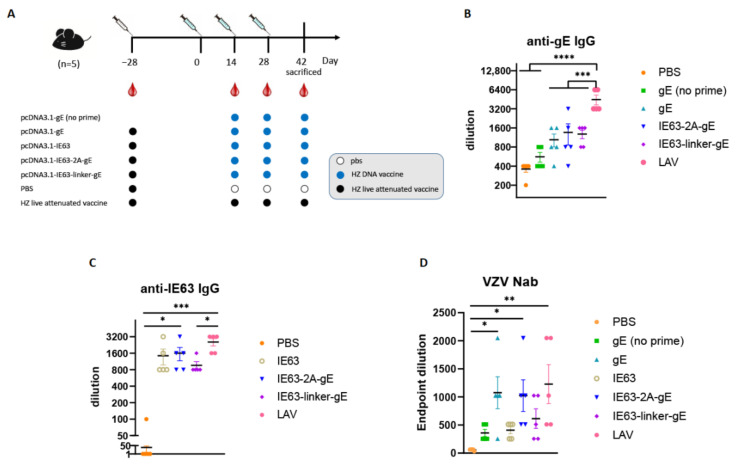
Specific humoral responses of HZ DNA vaccines in C57BL/6 mice. Six- to eight-week-old female C57BL/6 mice were subcutaneously immunized 1/10 human dose HZ live attenuated vaccine at day −28 (priming immunization), then intramuscularly immunized three times at day 0, day 14 and day 28 with 50 µg pcDNA3.1-gE, pcDNA3.1-IE63, pcDNA3.1-IE63-2A-gE, or pcDNA3.1-IE63-linker-gE, respectively, by in vivo electroporation (*n* = 5 for each group). The pcDNA3.1-gE (no prime) group did not recieve prime immunization with live attenuated vaccine but retained the three pcDNA3.1-gE immunizations. The blood was collected 2 weeks after the last immunization. (**A**) Immunization schedule. Anti-VZV gE antibody titers (**B**) and Anti-VZV IE63 antibody titers (**C**) were quantified by ELISA, respectively, from serum samples collected at day 42. (**D**) Neutralizing antibodies analyzed by FAMA. * *p* < 0.05; ** *p* < 0.01; *** *p* < 0.001; **** *p* < 0.0001; ns, no significant difference. Data represent mean ± SEM (*n* = 5).

Since most patients with HZ are VZV seropositive [33,34], mice, in this study, were inoculated with a live-attenuated herpes zoster vaccine 4 weeks before immunization to mimic the clinical setting. To confirm the immune responses, IgG and neutralizing antibodies were detected. Six groups of 6–8 weeks old C57BL/6J female mice were primed with a single 1/10 dose of VZV LAV (10^4.8^ PFU) and immunized with three doses of the indicated vaccine modalities delivered at 14-day intervals (Figure 2A). DNA encoding gE, IE63, IE63-2A-gE, and IE63-linker-gE was intramuscularly immunized in mice by in vivo electroporation according to the schedule presented in Figure 2A. Blood samples were collected from mice after two weeks of the last immunization. Serum samples from mice in the HZ live attenuated vaccine showed the highest gE- and IE63-specific IgG titers. The IE63-2A-gE and IE63-linker-gE groups induced slightly higher gE-specific IgG titers than the gE group (Figure 2B). The IE63-2A-gE and IE63 groups induced slightly higher IE63-specific IgG titers than the IE63-linker-gE group (Figure 2C). VZV-specific neutralizing antibodies were measured using the FAMA assay. The titers of IE63-2A-gE were similar to those of the gE and LAV groups (Figure 2D). The neutralizing titers of these three groups were slightly higher than those of IE63-linker-gE (Figure 2D), which illustrated that IE63 might not contribute to an increase in neutralizing ability. Taken together, the results indicate that HZ DNA vaccines induced VZV-specific humoral immune responses.

### 3.3. HZ DNA Vaccines Induces VZV-Specific IFN-γ+ T Cell Response in Mice

**Figure 3:**
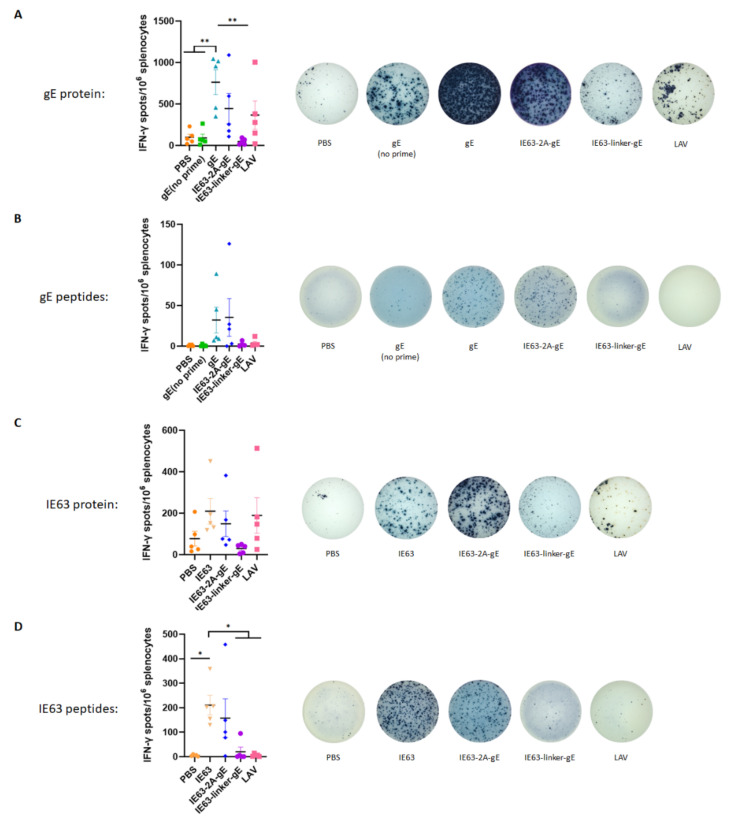
IFN-γ secreting cell frequencies in immunized mouse splenocytes. Frequencies of antigen-specific IFN-γ secreting cells were assayed via IFN-γ ELISPOT using freshly isolated splenocytes on day 42. The cells were stimulated with 5 μg/mL gE protein (**A**), IE63 protein (**C**) or 10 μg/mL gE peptides (**B**), IE63 peptides (**D**) for 24 h. The y axis shows the spot forming cells (SFCs) among 10^6^ splenocytes. Representative raw ELISPOT data were presented. * *p* < 0.05; ** *p* < 0.01. Data represent mean ± SEM (*n* = 5).

A strong VZV-specific CMI is required to prevent HZ, which is characterized by high IFN-γ secretion [9,35,36]. T cells are mainly responsible for IFN-γ secretion during adaptive immunity, which means that the level of IFN-γ produced by HZ vaccines could reflect their ability to elicit CMI. To investigate the VZV-specific CMI, the number of splenocytes secreting IFN-γ was detected by ELISPOT on day 42 by stimulation with gE protein, gE peptides, IE63 protein and IE63 peptides, respectively. As shown in Figure 3A, the gE group showed the highest gE-specific IFN-γ^+^ T cell frequencies, and the IE63-2A-gE group showed comparable INF-γ^+^ T cell frequencies with those of the LAV group. When splenocytes were stimulated with gE peptides, the IE63-2A-gE and gE groups showed comparably high INF-γ^+^ T-cell frequencies (Figure 3B). It was observed that the IE63-2A-gE group produced comparable levels of IE63-specific IFN-γ^+^ T cells to those of the IE63 or LAV group when stimulated with IE63 protein (Figure 3C). When splenocytes were stimulated with IE63 peptides, IE63-2A-gE and IE63 groups showed comparably high INF-γ^+^ T cell frequencies (Figure 3D). These data indicate that IE63-2A-gE induced a comparable VZV-specific IFN-γ^+^ T cell response with that of gE and IE63 in mice.

### 3.4. HZ DNA Vaccines Induces VZV-Specific Th1/Th2 Cytokine Secretion

**Figure 4:**
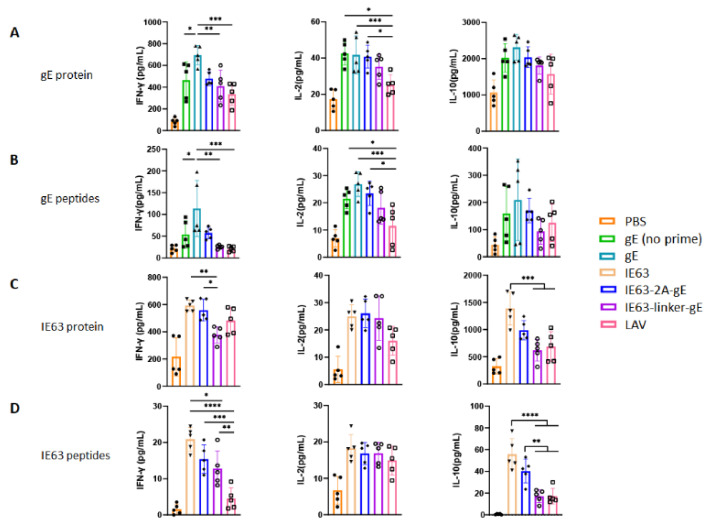
IFN-γ, IL-2, and IL-10 levels in immunized mouse spleen cells. The levels of Th1 (IFN-γ and IL-2) and Th2 (IL-10) cytokines secreted by mouse spleen cells stimulated with gE protein (**A**), gE peptides (**B**), IE63 protein (**C**), and IE63 peptides (**D**) were detected 14 days after the third vaccination. * *p* < 0.05; ** *p* < 0.01; *** *p* < 0.001; **** *p* < 0.0001; ns, no significant difference. Data represent mean ± SEM (*n* = 5).

Upon antigen stimulation, T cell precursors differentiate into Th0 cells, which further differentiate into Th1, Th2, or others. These cells produce various cytokines that promote or suppress the secretion of other cytokines through complex mechanisms. Th1 cells secrete IFN-γ, IL-2, and TNF-α, whereas Th2 cells secrete IL-4, IL-10, IL-13, and IL-17E/IL-15 [37]. To evaluate the immune responses elicited by HZ vaccines, the levels of IFN-γ, IL-2, and IL-10 secreted by spleen cells after gE or IE63 stimulation were detected by ELISA on day 14 after the third vaccination. When splenocytes were stimulated with gE protein or gE peptides, the gE group induced significantly higher IFN-γ levels than the IE63-linker-gE and LAV groups (Figure 4A,B). The IE63-2A-gE and IE63-linker-gE groups showed comparable levels of IFN-γ secretion to that of the LAV group (Figure 4A,B). The level of IL-2 secretion in splenocytes stimulated with gE protein or peptides in the IE63-2A-gE group was significantly higher than that in the LAV group (Figure 4A,B). There were no significant differences in IL-10 levels among the gE, IE63-2A-gE, IE63-linker-gE, and LAV groups (Figure 4A,B). When splenocytes were stimulated with IE63 protein, the IE63 and IE63-2A-gE groups showed increased IFN-γ levels compared to the IE63-linker-gE group (Figure 4C). No significant difference in IL-2 levels was observed among the vaccine groups (Figure 4C). The IE63 group showed higher levels of IL-10 than the IE63-linker-gE and LAV groups (Figure 4C). When splenocytes were stimulated with IE63 peptides, the IE63-2A-gE and IE63-linker-gE groups induced significantly higher IFN-γ levels than those in the LAV group (Figure 4D). When stimulated with IE63 peptides, the IE63, IE63-2A-gE, IE63-linker-gE, and LAV groups showed comparable levels of IL-2 secretion (Figure 4D). The IE63 and IE63-2A-gE groups showed significantly higher IL-10 levels than the IE63-linker-gE and LAV groups (Figure 4D). Taken together, IE63-2A-gE may be a better design due to a Th1-biased immune response.

### 3.5. Cytotoxic Activity of Splenocytes Derived from Immunized Mice

**Figure 5:**
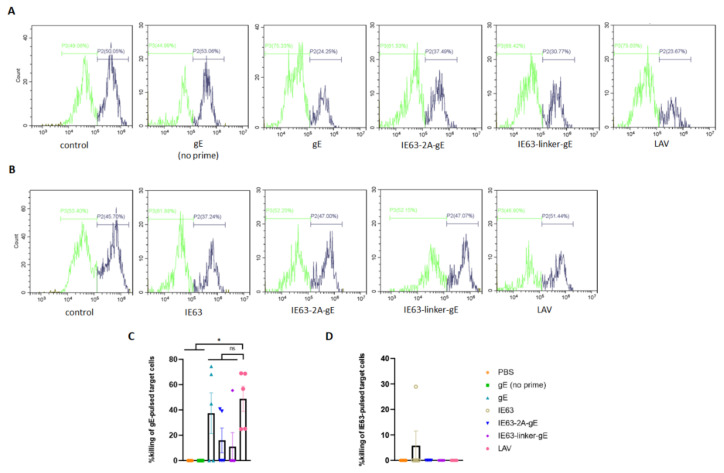
Cytotoxic activity of splenocytes derived from immunized mice. Antigen-specific cytotoxic activity of splenocytes derived from immunized mice on day 42 at effector-to-target ratios of 50:1. (**A**) Representative histograms of CFSE^low^ (unpulsed, green) and CFSE^high^ (gE pulsed, gray) populations, 8 h after incubation with splenoctyes from immunized mice. (**B**) Representative histograms of CFSE^low^ (unpulsed, green) and CFSE^high^ (IE63 pulsed, gray) populations, 8 h after incubation with splenoctyes from immunized mice. (**C**) Killing ratio of the gE-pulsed target cells. (**D**) Killing ratio of the IE63-pulsed target cells. * *p* < 0.05, ns, no significant difference. Data represent mean ± SEM (*n* = 5).

To determine the cytotoxic effect of splenocytes derived from mice immunized with HZ vaccines, we performed an in vitro cytotoxicity assay, in which equivalent numbers of antigen-pulsed and unpulsed target EL-4 cells were incubated with splenocytes isolated from immunized mice at effector-to-target ratios of 50:1 for eight hours. Flow cytometry analysis revealed that the killing ratio of the LAV group was significantly higher than that of the PBS and gE (no-prime) groups. The gE-specific CMI of the IE63-2A-gE group was lower than that of the gE and LAV groups, although no statistically significant difference was observed (Figure 5A,C). The IE63-specific cytotoxic effect of these groups was <10% and was similar among the five groups (Figure 5B,D). Unfortunately, weak cytotoxic T cell responses were observed in this experiment.

## 4. Discussion

Zostavax^®^ is a live attenuated vaccine that has been used since 2006; however, one limitation is that the rates of prevention decrease with increasing age. Additionally, it is contraindicated in immunocompromised people [38]. Shingrix^®^ is a recombinant subunit vaccine containing the AS01B adjuvant that exhibits long-term and high vaccine efficacy regardless of age. However, MPL and QS-21 are extremely expensive, which prevents their further application. Additionally, DNA vaccines highlight the advantages of precise construction, easy production, convenient transport, and satisfactory biosafety, which make them more easily used in developing countries.

VZV gE is the most abundant and immunogenic VZV glycoprotein and an important target for the host immune response. Therefore, VZV gE is widely used as an antigen for HZ vaccines to induce robust immune responses. IE63 could also represent a putative vaccine candidate because it is expressed very early during viral replication and is expressed during virus latency, which means that IE63-specific CMI might be conducive to clearing latently infected cells. Consequently, IE63 could be useful for the prevention of HZ in patients already infected with VZV. Accordingly, in this study, we designed four HZ DNA vaccines encoding VZV gE, IE63, IE63-2A-gE, and IE63-linker-gE to evaluate the immunogenicity of different antigen combinations of gE and IE63.

In this study, all DNA vaccines encoding gE induced comparable levels of gE-specific IgG titers in mice; all DNA vaccines encoding IE63 induced comparable levels of IE63-specific IgG titers. The LAV group showed significantly higher gE- and IE63-specific IgG titers than the other groups. However, C57BL/6J mice were primed with an LAV of 1/10 of human dose in this study, containing approximately 10^3.8^ PFU, which was three-fold higher than that used in other studies [33,34]. Additionally, for the LAV control group, VZV-primed animals were injected intramuscularly with a single 1/10 HD of LAV on day zero in other studies. However, mice in the LAV group were immunized three times in this experiment [33,34]. The neutralizing antibodies measured by FAMA of the gE and LAV groups were comparable to those of the IE63-2A-gE group, which was consistent with the results of the recgE–IE63 subunit vaccine tested in mice and guinea pigs [28]. The absence of anti-IE63 IgG responses to neutralize VZV (Figure 2D) could be explained by the fact that IE63 is a structural protein located in the viral tegument and is located primarily in nuclei of infected cells [39]. Other literature testing the IE63 subunit vaccine also found absent neutralizing antibody [28].

For HZ vaccines, CMI, rather than humoral immune responses, plays a key role in restricting VZV reactivation, which is vital for zoster vaccine efficacy [40]. Next, we found that DNA vaccines induced VZV-specific T-cell responses. IE63-2A-gE induced higher IFN-γ response than LAV (Figure 3 and Figure 4), and also induced higher IL-2 secretion than LAV in splenocytes when stimulated with gE (Figure 4A,B). IFN-γ and IL-2 activate Th1 cells, which elevate T cell activation, expansion, differentiation, and maintenance. IL-2-secreting immune cells also mediate the differentiation of CD8+ T cells into effector and memory T cells [41,42]. Based on these findings, IE63-2A-gE has the potential to enhance T cell responses in older patients, because cellular responses decline with age.

Furthermore, DNA vaccines that encode antigens result in intracellular presentation and trigger innate immune pathways, resulting in virus-like immune stimulation [29]. This vaccine type has the potential to efficiently stimulate CD8^+^ T cell responses with peptide presentation on MHC class I molecules. Unfortunately, in this study, weak cytotoxic CD8^+^ T cell responses were observed in HZ DNA vaccine groups. Previous studies indicated that anti-VZV T cell responses tend toward CD4^+^ T cell responses, especially with surface glycoproteins such as gE [34,43]. Furthermore, it has been found that IE63 protein-specific T cells are strongly dominated by CD4^+^ T cells [26]. Unfortunately, CD8^+^ T cell responses were rarely observed in this study, implying auxiliary treatments to boost VZV-specific CD8^+^ T cell responses of IE63-2A-gE DNA vaccine are probably helpful. Further tests of the co-administration of IE63-2A-gE DNA and cytokine DNA or adjuvants to enhance CD8^+^ T cell responses are needed [44,45].

The major goal of HZ vaccination is to induce strong VZV-specific T cell responses to prevent HZ and its associated complications. This study showed that the HZ DNA vaccine encoding IE63-2A-gE elicited comparable levels of neutralizing antibodies to those of the LAV group. The IE63-2A-gE group induced higher IFN-γ and IL-2 levels than the IE63-linker-gE and LAV groups and induced an IFN-γ-secreting cell frequency comparable to that of the gE or IE63 groups. Given that the component of IE63 plays an important role in eliciting a specific T cell response [25,26], the IE63-2A-gE DNA vaccine probably provides benefits. However, since VZV is a highly species-specific virus that induces clinical symptoms only in humans or non-human primate species, primed VZV will neither be latent nor reactivated in mice [46,47,48]. The protective efficacy of the IE63-2A-gE DNA vaccine for controlling viral reactivation cannot be evaluated in a mouse model. Further evaluations of IE63-2A-gE DNA vaccine efficacy in rhesus macaque models are required. Concludingly, this study provides novel insights into the immune mechanism of VZV vaccines and introduces IE63-2A-gE as a candidate antigen for HZ vaccine design.
